# Sex Influence on the Functional Recovery Pattern After a Graded Running Race: Original Analysis to Identify the Recovery Profiles

**DOI:** 10.3389/fphys.2021.649396

**Published:** 2021-03-18

**Authors:** Robin Macchi, Fabrice Vercruyssen, Arnaud Hays, Gaetan Aubert, Gaetan Exubis, Pascale Chavet, Emmanuelle Goubert, Robin Souron, Yoko Kunimasa, Caroline Nicol

**Affiliations:** ^1^Institute of Movement Sciences (ISM), National Center of Scientific Research (CNRS) & Aix Marseille Univ, Marseille, France; ^2^Impact of Physical Activity on Health (IAPS), University of Toulon, Toulon, France; ^3^Institut Carnot STAR, Aix Marseille Univ, Marseille, France; ^4^Osaka University of Health and Sport Sciences, Osaka, Japan

**Keywords:** sex influence, endurance running, functional recovery, fatigue, recovery profiles, multivariate analysis

## Abstract

This study investigated the sex influence on the acute and delayed fatigue effects of a 20 km graded running race. Eighteen recreational runners, 10 women and 8 men, completed the race. The testing protocol included five sessions: a week before the race (PRE), 35 ± 15 min after (POST), 2 h, 2 and 4 days (2D and 4D) later. Each session included uni- and bilateral maximal isometric voluntary contractions of the knee extensors (MVC), a squat jump (SJ), and a drop jump (DJ). Acute and delayed muscle soreness (DOMS) were evaluated for the quadriceps, hamstring and triceps surae muscle groups. The 2D and 4D sessions included also a horizontal force-velocity test (HF-V) performed under five resistive conditions. For each test, a set of key variables was computed to characterize the lower limb functional recovery. Mixed ANOVA analyses revealed significant (sex × time) interactions, with larger acute drops for men in MVCs and earlier recovery for women in the bilateral MVC (*p* < 0.001) and DJ (*p* < 0.05) tests. Only women reported DOMS for the hamstrings at 2D (*p* < 0.001) and showed small improvements in pure concentric SJ (*p* < 0.05) and HF-V (*p* < 0.01) tests at 4D. As expected, DOMS disappeared prior to the complete functional recovery. These results confirmed the combined influence of testing task and sex on the functional recovery pattern while supporting a lesser and faster recovery in women. The originality of this study lies in the complexity and sex-dependence of the functional recovery pattern revealed by a multiple factorial analysis which was used to identify the most discriminating tests and variables in the recovery pattern. The obtained clusters highlighted some recovery profiles associated with greater risks of injury when starting to run again. However, the lack of sex × time interaction for normalized values emphasizes the major influence of men’s initially higher functional values compared to women.

## Introduction

Women participation and/or performances among endurance running races (from 5 to 1,000 km) have increased over the last 25 years (Bam et al., 1997; Zingg et al., 2014; Joyner, 2017; Scheer, 2019). For instance, in 2019, Jasmin Paris won the 430 km Spine Race in 83 h. Breaking the previous male record (by 12 h), this female athlete demonstrated impressive qualities of both muscular endurance and recovery between the consecutive running phases of the race.

In endurance running exercises, the impact loads are repeated over time, stressing metabolic, mechanical, and neural components (Nicol and Komi, 2010). The repeated ground impact loads may vary from 2 to 3 times bodyweight, leading to subsequent eccentric muscle actions that are likely to induce reversible ultrastructural muscle damage with subsequent edema, remodeling/inflammation processes and delayed onset muscle soreness (DOMS) (Nicol et al., 2006; Nicol and Komi, 2010). Lower-limb muscles become weak, sore, stiff and occasionally swollen for a few days. DOMS is a subjective sensation of dull pain and discomfort that increases in intensity during the first 2 days, remaining symptomatic for 1–2 additional days before disappearing on days 5–7. It should be mentioned that neither the degree nor the timing of exercise-induced muscle damage per se correlates well with the changes in DOMS sensation (Nurenberg et al., 1992; Howell et al., 1993; Paulsen et al., 2012). The timing of DOMS disappearance is particularly important for injury prevention, as it usually occurs prior to complete structural and functional recoveries (Nicol and Komi, 2010), resulting in an overestimation of the actual functional capacities that could increase the risk of injuries by starting to run again too soon.

After endurance running exercises, it remains unclear whether sex differences exist in the functional recovery pattern. Mostly examined in men, the classical recovery pattern is usually considered as biphasic (Nicol et al., 2006; Nicol and Komi, 2010). First, this pattern is characterized by large acute drops in maximal voluntary contraction (MVC), maximal activation and stretch-reflex response leading to reduced maximal isometric as well as dynamic performances. This is followed by a short-term “acute” recovery within the next 2 h, which precedes “delayed” functional reductions 1–2 days later. Depending on the training status and on the exercise severity, the functional recovery may still require 1–2 weeks to be completed. Importantly, the functional recovery pattern appears to be testing task dependent. For instance, our earlier marathon studies (Nicol et al., 1991a, b) revealed larger acute functional decrements in maximal isometric knee extension test than in maximal sprint and drop-jump performances. In contrast, pluri-articular jump tests with no ground impact showed no significant decrement in performance. Furthermore, scientific evidence suggests that sex-based differences are specific to the involved muscle(s) and to the fatiguing task (contraction mode, intensity and velocity) (Hunter, 2016a) as well as to the testing task (Hill et al., 2018). This emphasizes the need for using a battery of varied tests to examine the sex-influence on the recovery pattern.

It is noteworthy that the recovery pattern after running for women has only been investigated by a few studies that are difficult to compare. Among expert runners, it has been reported that a 110 km ultra-trail race induced acute lower drops in maximal force of knee extensors and peripheral fatigue of plantar flexors in women compared to men (Temesi et al., 2015). In regularly trained recreational runners (>220 min or 50 km per week), it has been found that a half-marathon results either in a lower decline in maximal knee extension force in women than in men (Glace et al., 1998), or in similar functional deficits between them (Boccia et al., 2018) after the race. In the delayed recovery phase, young sportsmen and women are reported to present similar time course of structural recovery (Sorichter et al., 2001) and neuromuscular changes (Martin et al., 2004) after a 20–30 min downhill treadmill run. However, in young sedentary people, a given downhill exercise led to a shorter inflammatory process in women than in men (48 vs. 72 h) whereas DOMS remained elevated up to 72 h in women (Oosthuyse and Bosch, 2017). In these studies, the first 2-h post-exercise enabling the differentiation of the acute vs. delayed period has not been examined, thus the possibility to identify a biphasic recovery pattern for these populations remains to be clarified.

As recently reviewed by Hunter (2016a), both past and even recent studies on the underlying fatigue mechanisms generally involved men, or often lacked the sex-distinction in their set-up and discussion. Although women are usually characterized by lower muscle mass, force and power than men, women are often less fatigable than men at similar relative intensity of fatiguing isometric contractions (Hunter, 2014). Aside of a relatively greater proportional area of slow twitch muscle fibers, the lesser fatigability of women might be attributed to the combined direct and indirect influences of sexual hormones (estrogens in particular) on muscle perfusion and metabolism, leading to lesser production of metabolites, sensitization of muscle afferents and reduction in voluntary activation (Hunter, 2014, 2016a, 2016b). As reviewed by Enns and Tiidus (2010), sex and estrogen are reported as potentially attenuating indirect indices of exercise-induced skeletal muscle damage (such as DOMS and CK) by influencing the inflammation and repair processes.

This study was thus designed to characterize the women vs. men recovery pattern including measures within the first 2-h post-exercise and up to 4 days consecutively to a graded 20 km road race expected to induce muscle damage. It was first hypothesized that the functional recovery pattern would be both testing task- and sex-dependent, while occurring earlier in women. Our second hypothesis was that DOMS would be sex and muscle dependent while disappearing prior to the complete functional recovery. In addition, a multidimensional analysis that extracts the main information by reducing the dimensions while considering the different times of the recovery was deemed appropriate to further examine specific and/or descriptive aspects of the recovery pattern. In addition, a hierarchical clustering analysis was considered as the key analysis to identify the different recovery profiles after the exhaustive running race.

## Materials and Methods

### Participants

All participants were recreational runners who had registered 6 months earlier to take part to the international Marseille-Cassis race of 20 km including positive and negative gradients (+382 m and −294 m). Prior to the experiment, the sample size was calculated using G Power (Version 3.1.9.7). According to the experimental design (two between groups and five repeated measures) and for a medium effect size (i.e., η^2^ = 0.06), 10 participants in each group were required to obtain a statistical power of 80%. Due to the complexity in recruiting runners who could participate to the five testing sessions and the loss of two participants due to back pain and torn muscle during the race, the final sample size was slightly smaller. Thus, the final group of participants (Table 1) included 18 volunteers: 10 healthy females (age: 35 ± 7 years, body mass: 61.1 ± 11.4 kg, height: 1.66 ± 0.08 m) and 8 healthy males (age: 29 ± 7 years, body mass: 70.9 ± 6.2 kg, height: 1.76 ± 0.06 m). Three women were in the follicular phase, four in the luteal phase, and three were amenorrhoeic. All participants were recreational endurance runners, as inclusion criteria included running training for 4 h or less each week and not having participated more than once in the current running race. It is worth mentioning that they all trained mainly level running. The current study was approved by the local ethics committee and, in accordance with the Helsinki Convention, written informed consent was obtained from all runners.

**TABLE 1:** Group-mean (± standard deviation) of the participants’ characteristics, racing time, and testing performance values at the pre-race session (PRE).

	Men	Women	
Finishing time (hh:mm)	02:05:00 ± 0:15	02:08:00 ± 0:23	NS
% of winner’s time per sex	205 ± 24	188 ± 33	NS
Age (yr)	29 ± 7	35 ± 7	NS
Height (m)	1.76 ± 0.06	1.66 ± 0.08	*
Mass (kg)	70.9 ± 6.2	61.1 ± 11.4	NS
PRE MVC_*BLT*_ (N)	1,784 ± 469	906 ± 268	***
PRE MVC_*DL*_ (N)	1,022 ± 261	570 ± 143	***
PRE MVC_*NDL*_ (N)	959 ± 204	564 ± 115	***
PRE vto SJ (m/s)	2.71 ± 0.23	2.12 ± 0.20	***
PRE vto DJ (m/s)	2.28 ± 0.19	1.81 ± 0.23	***
PRE vto HF-V 0% BW (m/s)	2.7 ± 0.19	2.05 ± 0.17	***
PRE vto HF-V 20% BW (m/s)	2.42 ± 0.14	1.86 ± 0.17	***
PRE vto HF-V 40% BW (m/s)	2.22 ± 0.14	1.68 ± 0.14	***
PRE vto HF-V 60% BW (m/s)	1.87 ± 0.15	1.42 ± 0.13	***
PRE HF-V ISOM (N)	1,611 ± 201	1,040 ± 158	***

Maximal voluntary isometric tests of knee extension: bilateral (MVC_*BLT*_), and unilateral with the dominant leg (MVC_*DL*_) or the non-dominant leg (MVC_*NDL*_). Velocity at take-off (vto) in the horizontal force-velocity (HF-V) test at 4 different loads expressed in percentage of body weight (BW). Statistical sex difference: *p < 0.05, ***p < 0.001; NS: non-significant.

### Experimental Design

**FIGURE 1:**
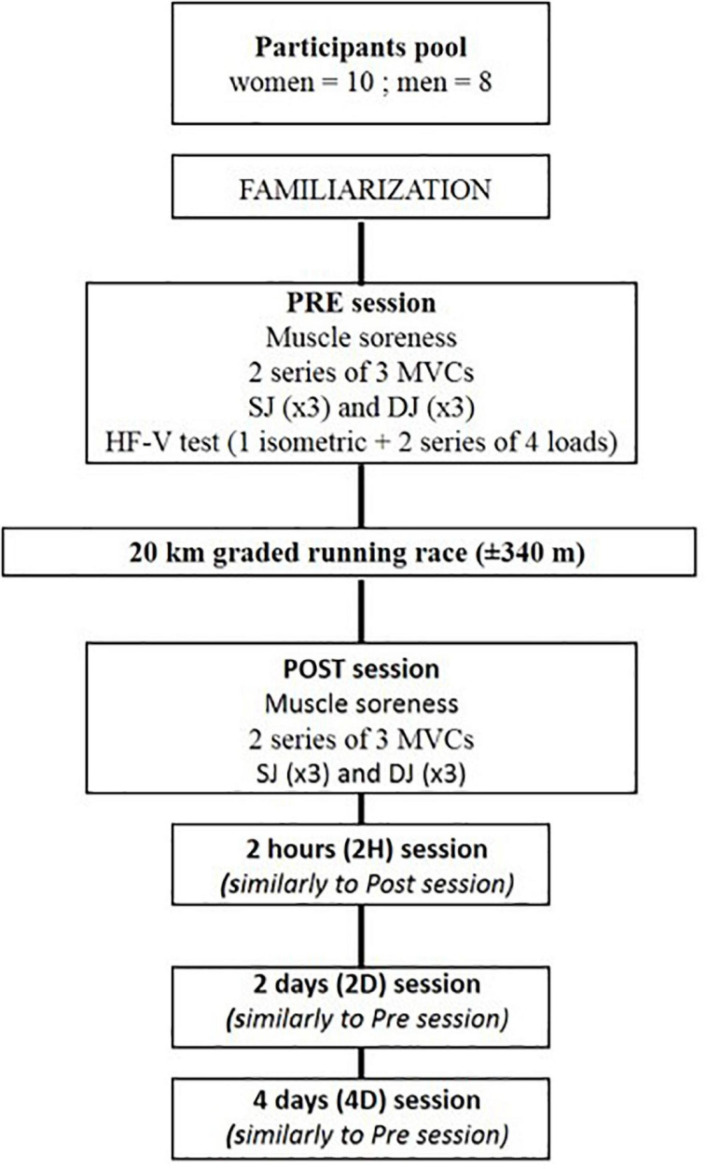
Experimental protocol. MVC, maximal voluntary isometric test of knee extension; HF-V, horizontal force-velocity test; SJ, squat jump; DJ, drop jump tests.

The testing protocol included five experimental sessions: a week before the running race (PRE), as quickly as possible (i.e., 35 ± 15 min) after the race (POST), 2 h (2H), 2 and 4 days later (2D and 4D, respectively) (Figure 1). The PRE session, which included a familiarization phase for each test, followed by a complete rest period dedicated to the medical interview before providing the control values for the following testing sessions. The PRE, 2D and 4D sessions lasted approximately 1.5 h including electromyographic (EMG) electrode placement, warm-up and the five tests. The POST and 2H sessions, which did not include the HF-V test and therefore did not require any EMG measurements, lasted only 15 min each, while including a warm-up at 2H but not at POST. Between these two measurement times, food and drinks were offered to all runners who could rest near the test room. All sessions began with a check of the runner’s body mass on a Tanita scale (MC980MA Tanita).

To assess lower-limb muscle soreness, runners had to step on and off a 50 cm high wooden box with the same lower limb. The test was repeated with the other limb. Visual analogic scale (0–10) was used to evaluate muscle soreness from the quadriceps, hamstrings, triceps surae and tibialis anterior muscles after each up- and downward movement.

Functional tests were preceded by a sex-standardized 10 min warm-up, except for POST session. The warm-up started by cycling for 6 min at 100 W for men and 75 W for women at a cadence ranging from 60 to 80 rpm (e.g., Hintzy et al., 2001). This was followed by two sequences that were repeated twice: 1 min at 50 W for men (40 W for women), and 30 s at 200 W for men (150 W for women) at a self-selected cycling cadence. Warm-up ended with an active recovery phase of 1 min at 40 W. Four maximal tests were performed in a given order: uni- and bilateral MVCs, three squat jumps (SJ) and three drop jumps (DJ) with 5–10 min of intermediate passive recovery. The PRE, 2D and 4D sessions included also a horizontal force-velocity (HF-V) test.

### Measurement Protocol

The knee MVC tests included two series of three MVCs performed on a knee extension ergometer (S2P Ltd, Ljubljana, Slovenia, EN 60204-1) with the arms crossed on the chest. Each series started with the unilateral MVC test with the dominant lower limb (MVC_*DL*_), followed by the non-dominant (MVC_*NDL*_) and the bilateral one (MVC_*BLT*_). The series and the trials were separated by 2 min and 30 s of passive recovery, respectively. To secure the repeatability of the testing position, the hip and knee flexion angles were set at 100° and the leg lever arm was individually adjusted at the same position between sessions. Participants were verbally encouraged and instructed to develop their force as fast as possible and to keep on producing maximal effort until the scheduled stop 3 s later. The produced torque was recorded at 1 kHz and the corresponding force was computed using a calibration matrix determined for the left and right lever arms of the ergometers.

The SJ test consisted of three maximal trials (with a 30 s intermediate passive recovery) performed from a static position at 90° knee angle and with the arms held akimbo. To record the potential fatigue effect on the runner’s proprioception, their self-adopted starting position was recorded, but each trial was then performed from the position at PRE. For this purpose, a laser (CL2i STANLEY) was used to quantify the vertical height of a passive reflective marker set on the left greater trochanter. Countermovement was prohibited and additional trials were performed when the criteria were not met.

The DJ test consisted of three maximal trials (with 1 min intermediate passive rest) from their optimal dropping height (set at either 30, 40, or 50 cm) previously checked during the familiarization phase. They were instructed to keep the arms akimbo, look straight ahead at a visual cue on the wall before dropping down and rebounding with maximal effort just after ground contact (Komi and Bosco, 1978), with minimal knee flexion while avoiding touching the floor with the heel (Cappa and Behm, 2013). When all requirements were not met, the trial was repeated.

The HF-V test consisted of two trials of ballistic squat jumps performed in a supine position on a frictionless sled (INPI deposit n°: FR2011204) under five resistive conditions: 0, 20, 40, and 60% bodyweight (BW) as well as in an isometric (ISOM) condition similarly to the tests used by Meylan et al. (2015). In all testing conditions, the starting position was held still for 2–3 s with hip, knee and ankle joints at 90°. The isometric test was always performed at first while the dynamic conditions were performed in a randomized order among the subjects. For a given runner, the order of the testing conditions remained the same in each session. They were asked to apply their force as rapidly as possible on the force plates to reach the highest velocity at the end of the push-off. Countermovement was verbally forbidden and carefully checked; the trial being repeated if any. Recoveries of 30 s and 180 s were provided between trials and between loads, respectively.

For all jump tests, two Kistler force plates [SJ and DJ: Kistler 9287CA (0.9 × 0.6 m); HF-V: Kistler 9260AA3 (0.5 × 0.3 m)] were used to record the normal component of the ground reaction forces produced under each foot. The sampling frequency was set at 1.5 kHz.

### Data Analyses

The pacing strategy was obtained from the running time records provided by the organizers every 5 km of the race. The subjective rate of perceived exertion was evaluated after the race at the POST session using a 6–20 Borg scale (Borg, 1970).

For the knee MVC tests, maximal torque was the highest peak torque of the two trials for uni- and bilateral conditions. The obtained torque values were expressed as force values using the calibration matrix and the peak force (F_*max*_) calculated. The absolute asymmetry index (SI) was computed as follows:

$$\text{SI}=100\,\left(\frac{\left|\mathrm{Ldom}-{\text{Lnon}}_{\text{dom}}\right|}{\text{avg}\,\left(\text{Ldom},\mathrm{Lno}\,{\text{n}}_{\text{dom}}\right)}\right)\,\left(\text{Zifchock et al., 2008}\right),$$

where Ldom and Lnon_dom_correspond to the dominant and non-dominant lower limbs, respectively. The non-absolute asymmetry index was also computed.

For the SJ and DJ tests, only the best of the three trials (selected from the velocity at takeoff) was considered for post-processing. All calculations were subsequently based on the vertical component of the ground reaction forces considered as the gold standard computation method (Attia et al., 2017). The instantaneous vertical velocity was obtained during the push-off phase by integrating the vertical acceleration of the center of mass (a) over time (Samozino et al., 2008):

$$\text{a}\,\left(\text{t}\right)=\frac{\mathrm{GRF}\,\left(t\right)}{\text{m}}-g$$

where “GRF” refers to the vertical component of the ground reaction force, “m” to the runner’s body mass and “g” to the gravity acceleration.

For the DJ test, the impact peak force (IPF) was also measured. The contact time was divided into its braking and push-off phases based on the velocity signal obtained from the GRF (Prieske et al., 2013). This led to the calculations of the braking phase duration (t_*brake*_) and mean force ($\bar{F}$_*brake*_). The respective push-off phase analysis included for both SJ and DJ tests: the mean force ($\bar{F}$_*po*_), velocity ($\bar{V}$_*po*_) and power ($\bar{P}$_*po*_) calculations. Both vertical velocity at take-off (vto) and maximal power output (P_*max)*_ were calculated (Morin et al., 2019). To analyze the potential discrepancy between these last two parameters due to a modified technique, vertical push-off time (tpo) and push-off distance (hpo = tpo × $\bar{V}$_*po*_) were calculated. The SI between the two legs was calculated from the vertical mean force values. For both jumps, force and power were normalized by body mass.

For the HF-V test, the horizontal displacement over time was recorded with an accuracy of 0.1 cm, using a linear encoder (Micro-Epsilon WDS-3000-P115-SR-U) attached to the sled. This signal was used to calculate the horizontal velocity. For each resistive load, the trial with the highest vto value was kept for post-processing. In the ISOM condition, the rate of force development (RFD) was calculated between 20 and 80% of the peak force. The subsequent push-off phase analysis was performed similarly than the one used for the SJ test.

### Statistical Analyses

Statistical analyses were performed with JASP (Version 0.10.2) and (R v3.6.3, R Core Team, 2020, R Foundation for Statistical Computing, Vienna, Austria). All data are presented as mean ± standard deviation (SD). One-tailed independent *t-*tests were performed to determine sex differences in the racing time, subjective rate of perceived exertion, and weight loss after the race (at POST). Shapiro–Wilk test was used to verify data normality based on the residuals. If not met, Mann-Whitney U test was used.

To test the sex-time fatigue effect, mixed two-way repeated-measures ANOVAs for time (PRE-2D-4D for the HF-V test; PRE-POST-2h-2D-4D for the other tests), with sex as a between-subject factor, were performed for each functional variable and each muscle soreness. For the pacing strategy, the time factor included the four successive sections of 5 km of the race. For the HF-V test, a mixed three-way repeated-measures ANOVAs was performed as the load factor included four load levels. Due to large inter-group differences at PRE in force, velocity and power, additional ANOVAs were performed after normalizing the data within each sex group by the group-mean PRE values. If a main time effect was found, Holm’s *post-hoc* test was used to assess the changes as compared to PRE. If a sex × time interaction effect was found, planned contrasts were used to test the time effect (as compared to PRE) for each sex group and the sex effect at each given time. This analysis avoided examining all possible comparisons, which increases the risk of error (Bartlett, 2017). Mauchly tests preceded each ANOVA analysis to verify sphericity. If the assumption of sphericity was violated, an epsilon Greenhouse-Geisser correction was applied. Each ANOVA was performed after checking for distribution normality with Shapiro-Wilk test. If not met, a simulation of 10.000 data sets representing the asymmetric distribution observed with groups having similar means was performed. If the obtained type 1 error exceeded 6%, a non-linear transformation of logarithm was applied to restore normality (Sainani, 2012). The alpha level was set at 0.05. Effect size was computed using Cohen’s *d* coefficient (Cohen, 1998) computed as the *t*-value standardized to the root mean square of the number of participants (Rosenthal, 1991). The effect size magnitude (ES) was then assessed using the following thresholds: <0.2, 0.2 to <0.6, 0.6 to <1.2 and 1.2 to <2.0 for trivial, small, moderate and large effects, respectively (Hopkins et al., 2009).

To globally analyze the dataset and to reveal the interactions between the absolute changes of all variables as compared to PRE, a multiple factor analysis (MFA) with FactoMineR and Factoshiny R-packages (Lê et al., 2008) was used, in which the time sessions represented the different groups. Such a subdivision could not have been performed by using a principal component analysis (PCA) (Pagès, 2014). The correlations between the different sessions were assessed by the RV coefficient (bounded between 0 and 1) which represents the link between two groups of variables based on inertia and normalized by the dimensionality of each group. All data were centered and reduced (corresponding to Z score) due to the non-equality of most variables. Sex and other descriptive variables (such as age, body mass, height, muscle soreness and racing time) were set in a supplementary group, which was only projected on the MFA dimensions. An ascendant hierarchical clustering on principal components was then performed to construct recovery profiles corresponding to functional responses to the race-induced fatigue.

## Results

Weather conditions varied slightly along the race, reaching a maximal temperature of 21°C with no wind. No significant difference in racing time was found between men and women (2:05 ± 0:15 and 2:08 ± 0:23, respectively). Although there was no significant difference in relative performance (i.e., relative to the fastest runner for each sex), women tended to be slightly faster than men (Table 1). Similarly, no significant differences were found between men and women in the pacing strategy that was mostly influenced by the graded profile of the race, the subjective perception of effort after the race (16.3 ± 2.2 and 15.7 ± 2.6, respectively), as well as the variation of body mass at POST (2.9 ± 1.2% and 2.1 ± 1.4%, respectively) or between sessions.

**FIGURE 2:**
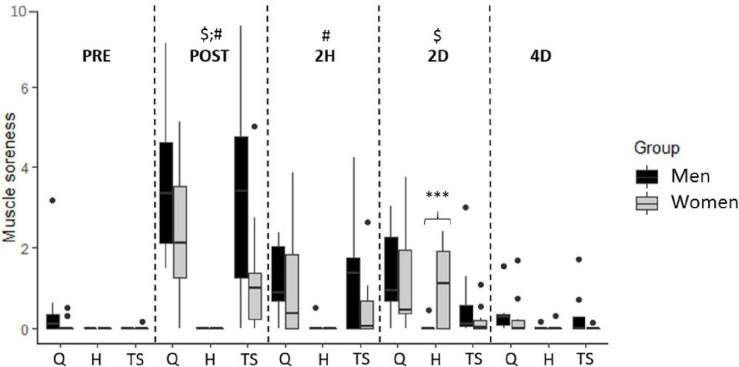
Muscle soreness reported by men and women for the quadriceps (Q), hamstring (H), and triceps surae (TS) muscle groups as a function of time. Outliers are indicated by dots. Significant time effect as compared to PRE for Q ($) and TS (#). ^∗∗∗^Sex-dependent difference for H (*p* < 0.001).

Since muscle soreness did not differ between dominant and non-dominant lower limbs, all subsequent analyses were performed on the mean values of the lower limbs for this parameter (Figure 2). A significant interaction effect was found (*p* < 0.001), reporting higher DOMS in women for the hamstring muscle group at 2D (*p* < 0.001, *ES* = 1.1). Independently of sex, a significant time effect was found for the quadriceps (*p* < 0.001) and triceps surae (*p* < 0.001) muscle soreness. Holm’s *post-hoc* showed increased muscle soreness at POST (*p* < 0.001, *ES* = 1.4) and 2D (*p* < 0.05, *ES* = 0.7) for the quadriceps while at POST (*p* < 0.01, *ES* = 1.0) and 2H (*p* < 0.05, *ES* = 0.7) for the triceps surae muscle group.

### Mixed Anova Analyses on the Functional Variables

**FIGURE 3:**
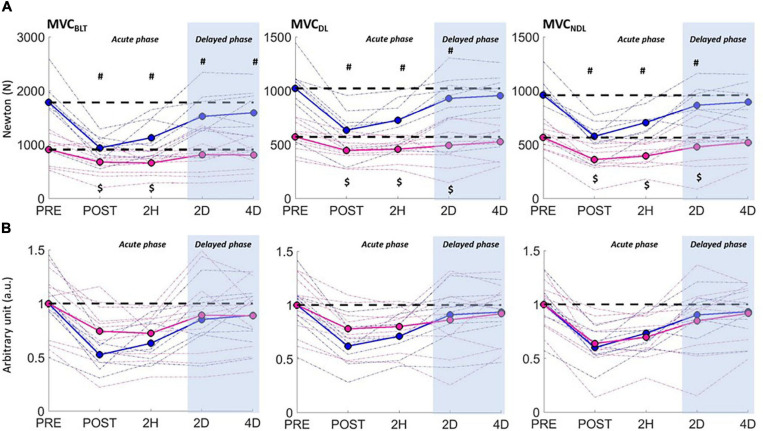
Maximal voluntary isometric contraction of knee extensors as a function of time for men (blue curve) and women (pink curve) in the bilateral (MVC_*BLT*_), the dominant (MVC_*DL*_), and non-dominant (MVC_*NDL*_) unilateral tests. Performances are expressed in absolute values **(A)** and in individually and group-mean normalized values **(B)**, with significant time differences (compared to PRE) showed for men (#) and women ($) (*p* < 0.05).

When focusing on the MVC tests (Figure 3 and Table 2), a main time effect was found and reflected large acute changes (i.e., at POST and 2H), followed by moderate delayed ones in each test. A moderate increase in the absolute SI was found at POST in the MVC_*BLT*_ (+24.1%, *p* < 0.05, *ES* = 1.1). A sex × time interaction effect was also found for each testing condition (BLT, *p* < 0.001; DL, *p* < 0.001; NDL, *p* < 0.05) on the absolute, but not on the normalized MVC values. For the MVC_*BLT*_, planned contrasts revealed large acute decreases for men (POST: *ES* = 2.3, 2H: *ES* = 1.8) and moderate ones for women (POST: *ES* = 0.7, 2H: *ES* = 0.8), so that the sex groups did not differ in absolute MVC_*BLT*_ at POST (p = 0.123). At 2D and 4D, only men showed delayed moderate (*ES* = 0.7) and small (*ES* = 0.5) decreases, respectively. For the unilateral tests, regardless of sex, the MVCs were reduced up to 2D. Men showed large acute drops, at POST (DL: *ES* = 2.3; NDL: *ES* = 2.1) and 2H (DL: *ES* = 1.8; NDL: *ES* = 1.4), but small ones at 2D (DL and NDL: *ES* = 0.5). Women showed mostly moderate acute drops, at POST (DL: *ES* = 0.8; NDL: *ES* = 1.3) and 2H (DL: *ES* = 0.8; NDL: *ES* = 1.1), and small ones at 2D (DL and NDL: *ES* = 0.5).

**TABLE 2:** Relative changes (as compared to PRE) in the bilateral (BLT), dominant leg (DL), and non-dominant leg (NDL) conditions of the maximal voluntary isometric test of knee extension.

	POST	2H	2D	4D
**BLT (%)**				
Men	−47 ± 8***	−35 ± 18***	−15 ± 11**	−11 ± 14*
Women	−26 ± 19**	−28 ± 9**	−14 ± 18	−13 ± 14
All	−37 ± 14***	−32 ± 14***	−15 ± 20**	−12 ± 14*
**DL (%)**				
Men	−40 ± 10***	−25 ± 17***	−10 ± 8*	−6 ± 8
Women	−37 ± 20***	−31 ± 14**	−18 ± 26*	−10 ± 12
All	−39 ± 15***	−28 ± 16***	−14 ± 17**	−8 ± 10*
**NDL (%)**				
Men	−39 ± 11***	−28 ± 13***	−10 ± 6*	−6 ± 5
Women	−22 ± 10***	−19 ± 11***	−15 ± 23*	−9 ± 11
All	−31 ± 10***	−24 ± 12***	−13 ± 15**	−8 ± 8*

*P < 0.05, **P < 0.01, and ***P < 0.001.

The pure concentric SJ and HF-V tests were not associated with any significant change in performance (vto) along the 4D recovery period. However, a main time effect was found in SJ for the entire group in $\bar{F}$_*po*_ (*p* < 0.05) due to a small increase at 4D (*p* < 0.05, *ES* = 0.2). In the HF-V test, independently of the load, a significant interaction effect (sex × time) was found for P_*max*_ (*p* < 0.05) and $\bar{F}$_*po*_ (*p* < 0.05), due to moderate increases of P_*max*_ (+9.9%, *p* < 0.001, *ES* = 1) and$\bar{F}$_*po*_ (+3.6%, *p* < 0.01, *ES* = 0.7) for women at 4D.

**FIGURE 4:**
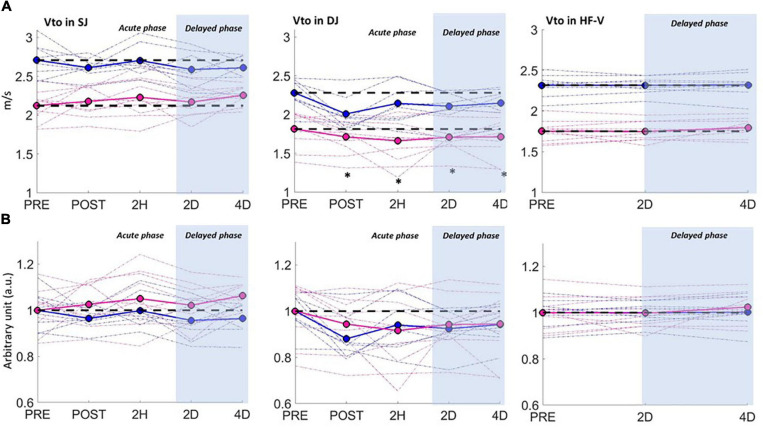
Pluriarticular dynamic test performances (vto: velocity at take-off) as a function of time for men (blue curve) and women (pink curve) in the squat jump (SJ), drop jump (DJ), and horizontal force-velocity (HF-V) tests. Performances are expressed in absolute values **(A)** and normalized values, with the significant time changes compared to PRE indicated for the entire group (^∗^) (*p* < 0.05). Due to the absence of significant interaction, the normalized values **(B)** are shown for each sex group with no statistics.

Analysis of the DJ test revealed a main time effect for the entire group that reflected large to moderate acute changes in most variables, followed by moderate to small ones at 2D and 4D (Figure 4 and Table 3). A sex × time interaction effect was found for P_*max*_ (*p* < 0.05), as men presented large decreases at POST (*ES* = 1.5) and moderate ones from 2H to 4D (*ES* = 1) while women showed only acute moderate decreases (POST: *ES* = 0.6; 2H: *ES* = 0.5). No interaction effect was found for the P_*max*_ data once normalized. The recovery of $\bar{F}$_*po*_ was found to be leg-dependent, the decrease being observed at POST only for NDL (*p* < 0.001, *ES* = 1.1) while at both POST (*p* < 0.001, *ES* = 1.5) and 2H (*p* < 0.01, *ES* = 0.9) for DL.

**TABLE 3:** Relative changes (Δ% as compared to PRE) for men and women for the braking and push-off phase parameters in the drop jump test: impact peak force (IPF), braking phase duration (t_*brake*_), push-off duration (tpo) and height (Hpo), mean force ($\bar{\text{F}}$), velocity ($\bar{\text{V}}$), and power ($\bar{\text{P}}$) of the braking (brake) or push-off (po) phase.

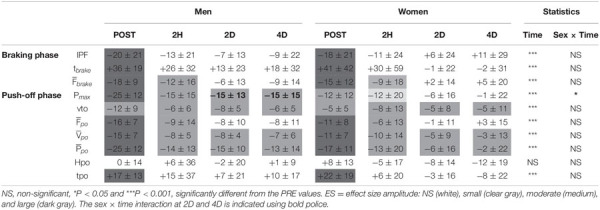

### The MFA Analysis

**FIGURE 5:**
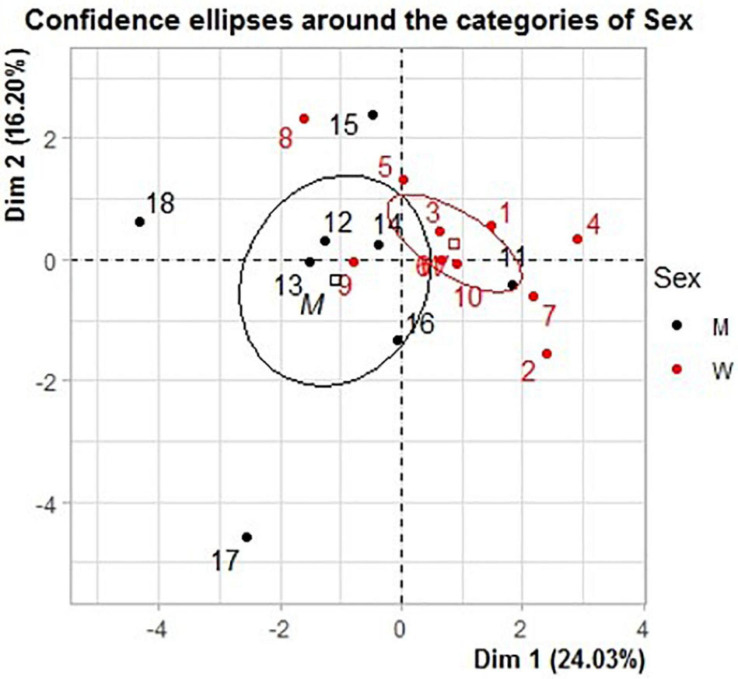
Results of the multiple factor analysis (MFA) with confidence ellipses around the sex groups. The first axis represents essentially the functional decrements in drop jump as compared to PRE. A high value on this axis indicates a limited drop in performance. The second axis represents mostly the decrements in the squat jump and horizontal force-velocity tests. A high value on this axis indicates a limited drop. The detailed variables are shown in Supplementary Table S1.

The two principal dimensions of the MFA on the individual changes are shown on Figure 5. The first dimension, which explained 24.03% of the variance, was mainly correlated to the acute and delayed functional decrements in DJ (such as decreases in force, velocity and power as well as increases in braking and push-off times), and the acute drops in MVC tests, notably for MVC_*BLT*_ and MVC_*DL*_ (*r* = 0.72, *p* < 0.001), and also for MVC_*NDL*_ (*r* = 0.56, *p* < 0.05). A significant correlation was found between sex and the first dimension (*r* = −0.54, *p* < 0.05). Most women displayed moderate or higher values on this dimension, reflecting their lower functional reductions in DJ than men. The second dimension, explaining 16.20% of the variance, was mainly represented by the functional SJ and HF-V variables, which generally varied in the opposite way to the DJ ones. The 16 best explanatory variables correlated with each dimension are shown in Supplementary Table S1.

The MFA analysis of the inter-session relationships revealed the highest correlations between sex and the functional changes at POST (RV coefficient = 0.41) and 4D (RV coefficient = 0.31) (Supplementary Table S2). The first dimensions of the POST and 4D sessions were highly correlated to the first dimension of the MFA (*r* = 0.91 and *r* = 0.77, respectively), and thus mainly explained by the DJ and MVC changes. Women presented higher values compared to men on the first dimension at POST (men = −1.34, women = 1.63) and 4D (men = −1.26, women = 1.01). This reflected their lower functional decrements in the DJ and MVC tests at POST, and their remaining functional decrements in the DJ test at 4D while showing an increased HF-V performance.

**FIGURE 6:**
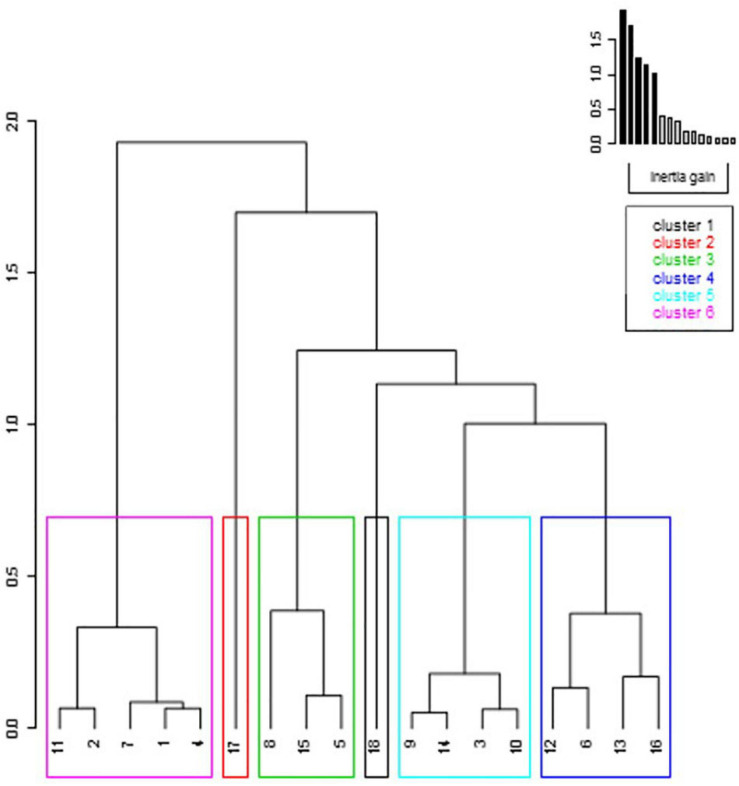
Cluster dendrogram based on the functional recovery variables. The detailed variables are shown in Table 4. The numbers 1–10 represent the women while the numbers 11–18 the men.

A*nalysis of the ascending hierarchical clustering with five dimensions* (representing 66.6% of the variance) identified six clusters. The dendrogram revealed globally two parts: one including the cluster 6 and a second one with the five other clusters (Figure 6). These were chosen from the inertia gain between the number of clusters. When considering the best explanatory variables of each cluster (Table 4), cluster 1 included only one runner, who presented large acute fatigue effects in SJ and delayed ones in DJ. Cluster 2 included the only runner who showed large delayed decrements in the HF-V and SJ tests. Cluster 3 included three runners showing large acute decrements in DJ, but improvements in SJ. Cluster 4 included four runners with decrements in HF-V and DOMS at 4D and low racing effort perception. Cluster 5 included the four runners showing slight drops in SJ, but slight improvements in the HF-V test (in particular for the ISOM condition) at 2H and 2D. Cluster 6 included the five runners who showed either no delayed decrease or even a slight improvement in DJ.

**TABLE 4:** Clusters of the hierarchical clustering analysis.

Cluster 1 [Men = 1 (100%)]				Cluster 2 [Men = 1 (100%)]				Cluster 3 [Men = 1 (33.3%); Women = 2 (66.7%)]			
	Category	Overall	*P*-value		Category	Overall	*P*-value		Category	Overall	*P*-value
	(%)	mean (%)			(%)	mean (%)			(%)	mean (%)	
**POST/PRE**				**2D/PRE**				**POST/PRE**			
$\bar{\text{P}}$po_SJ	–32.2	0.4	<0.01	tpo_HF-V_20%	22.5	2	<0.01	$\bar{\text{F}}$brake_DJ	–22.5	–13.1	<0.05
$\bar{\text{V}}$po_SJ	–29.2	–1.4	<0.05	tpo_HF-V_0%	16.7	4.1	<0.05	tbrake_DJ	90.8	38.6	<0.01
$\bar{\text{F}}$po_SJ	–13.1	0.4	<0.05	$\bar{\text{V}}$po_HF-V_20%	–30.2	–1.7	<0.001	tpo_DJ	44	19.6	<0.01
**2H/PRE**				$\bar{\text{P}}$po_HF-V_20%	–41.6	–2.2	<0.001	**2H/PRE**			
MVC_*DL*_	–42.1	–23	<0.01	$\bar{\text{V}}$po_HF-V_60%	–23	–2.7	<0.01	$\bar{\text{V}}$po_DJ	–25.8	–8.9	<0.01
hpo_DJ	86.4	–0.3	<0.01	$\bar{\text{P}}$po_HF-V_60%	–32.6	–2.8	<0.01	vto_DJ	–20.8	–7.2	<0.05
tpo_DJ	93.7	10.2	<0.05	$\bar{\text{F}}$po_HF-V_20%	–14.1	–0.3	<0.01	$\bar{\text{P}}$po_DJ	–13.7	–39.5	<0.05
**2D/PRE**				**4D/PRE**				$\bar{\text{F}}$brake_DJ	–10.3	–3.8	<0.05
hpo_DJ	38.1	–5	<0.05	tpo_HF-V_60%	31.9	2.9	<0.01	IPF_DJ	–42.5	–12.2	<0.05
$\bar{\text{F}}$po_DJ	–25.9	–4.2	<0.05	$\bar{\text{F}}$po_HF-V_60%	–17.8	0.2	<0.01	$\bar{\text{F}}$po_DJ	–7.4	–24	<0.05
**4D/PRE**				$\bar{\text{V}}$po_HF-V_60%	–25.8	–2.3	<0.001	tbrake_DJ	107	27.8	<0.01
IPF_DJ	–50.6	1.7	<0.01	$\bar{\text{V}}$po_HF-V_20%	–22.1	–1.1	<0.001	hpo_SJ	30.8	3.5	<0.01
Pmax_DJ	–44.3	–7.1	<0.01	$\bar{\text{P}}$po_HF-V_20%	–28	0.1	<0.01	**2D/PRE**			
$\bar{\text{F}}$brake_DJ	–36.5	–1	<0.01	$\bar{\text{F}}$po_HF-V_40%	19.3	1.9	<0.05	hpo_SJ	33.7	–5.4	<0.01
Pmax_SJ	–20.5	5.8	<0.05	Pmax_HF-V_40%	11.8	2	<0.05	tpo_SJ	36.4	–3.4	<0.05
$\bar{\text{P}}$po_DJ	–37.8	–6.5	<0.05	$\bar{\text{P}}$po_HF-V_60%	–32.2	–1.2	<0.001	vto_SJ	14.1	–0.3	<0.05
$\bar{\text{F}}$po_DJ	–29	–2.2	<0.05					**4D/PRE**			
								hpo_SJ	35.3	–2.8	<0.05
								tpo_SJ	22.7	–7.1	<0.05
								MVC_*DL*_	2.5	–7.7	<0.05

**Cluster 4 [Men = 3 (75%); Women = 1 (25%)]**				**Cluster 5 [Men = 1 (25%); Women = 3 (75%)]**				**Cluster 6 [Men = 1 (20%); Women = 4 (80%)]**			
	**Category**	**Overall**	***P*-value**		**Category**	**Overall**	***P*-value**		**Category**	**Overall**	***P*-value**
	**(%)**	**mean (%)**			**(%)**	**mean (%)**			**(%)**	**mean (%)**	

**2D/PRE**				**2D/PRE**				**POST/PRE**			
$\bar{\text{P}}$po_HF-V_40%	–9	1.1	<0.05	RFD_HF-V	174.2	26.9	<0.01	$\bar{\text{F}}$brake_DJ	–5.9	–16.5	<0.01
Pmax_HF-V_20%	–4.8	1.1	<0.05	Pmax_HF-V_0%	19.2	4.3	<0.01	**2H/PRE**			
$\bar{\text{V}}$po_HF-V_40%	0.7	–5.1	<0.05	vto_HF-V_0%	11.2	2.1	<0.01	tpo_DJ	–8.9	10.2	<0.05
Pmax_HF-V_0%	–5.4	4.3	<0.05	Fmax_HF-V_ISOM	17	0.1	<0.05	tbrake_DJ	–11.6	27.8	<0.05
vto_DJ	–11.4	–6.4	<0.05	**4D/PRE**				**2D/PRE**			
**4D/PRE**				vto_HF-V_0%	12.3	2.8	<0.01	tbrake_DJ	–21.5	5.4	<0.001
$\bar{\text{V}}$po_HF-V_40%	–5.1	1.5	<0.01	RFD_HF-V	124.1	19.3	<0.01	$\bar{\text{P}}$po_DJ	8.6	–9.6	<0.01
$\bar{\text{P}}$po_HF-V_40%	–7.4	2.9	<0.01	Pmax_HF-V_0%	28.1	9.9	<0.05	tpo_DJ	–15.2	1.8	<0.05
RFD_HF-V	–33.1	19.3	<0.05	hpo_HF-V_60%	6.3	–0.5	<0.05	$\bar{\text{F}}$brake_DJ	15.7	–1.7	<0.01
Effort perception (a.u.)	13.8	15.9	<0.05	$\bar{\text{P}}$po_HF-V_0%	18.7	2.8	<0.05	$\bar{\text{V}}$po_DJ	2	–6.3	<0.01
DOMS_4D (a.u.)	4.85	1.98	<0.05	tpo_HF-V_20%	6.6	–0.1	<0.05	$\bar{\text{F}}$po_DJ	9.3	–4.2	<0.01
								**4D/PRE**			
								$\bar{\text{F}}$po_DJ	17.1	–2.2	<0.001
								$\bar{\text{F}}$brake_DJ	24.9	–1	<0.001
								hpo_DJ	–19	–5.9	<0.05
								$\bar{\text{P}}$po_DJ	19.6	–6.5	<0.01
								tbrake_DJ	–31.2	7.2	<0.001
								tpo_DJ	–24.5	0.2	<0.01
								$\bar{\text{V}}$po_DJ	7.1	–4.9	<0.01
								Pmax_DJ	19.6	–6.5	<0.01
								Masse (kg)	55.5	65.5	<0.05
								Taille (m)	1.62	1.70	<0.05

**The overall mean corresponds to the averaged relative difference for the whole group as compared to the PRE session. Impact peak force (IPF); vertical push-off distance (Hpo), duration (tpo), and velocity at take-off (vto) in squat jump (SJ); maximal power (P_*max*_); mean force (*$\bar{F}$*), velocity (*$\bar{V}$*), and power (*$\bar{P}$*) of the braking (brake) or push-off (po) phase in drop jump (DJ) or in horizontal force-velocity (HF-V) test at given resistive loads set from 0 to 60% bodyweight; Delayed onset muscle soreness (DOMS).**

## Discussion

This study aimed to assess the sex influence on the functional recovery pattern after a 20 km graded road endurance race. The racing time (relative and absolute), subjective rate of perceived exertion and pacing strategy being similar between women and men, the functional results have been compared independently of these race parameters.

### Testing Task- and Sex-Dependent Effects on the Functional Recovery

Supporting our hypothesis that the functional recovery pattern would be testing-task dependent, the MVC and DJ tests revealed large acute performance decreases followed by moderate ones up to 4D whereas the concentric jump (i.e., SJ and HF-V) performances did not change significantly. Also confirming our expectations, significant sex × time interaction effects were found in all tests that provided for women indices of lesser functional decrements at POST and earlier recovery completeness than men. To allow comparison with the scientific literature that mostly used a unilateral MVC test to evaluate running-induced fatigue in men, the results of the MVC tests are discussed prior to the analysis of the other test results and/or sex × time interactions.

Following the 20 km graded road race, the MVC tests revealed mean decreases of 42 and 28% for men and women, respectively at POST, which are close to those observed after a 110-km walking/running ultra-trail with a ∼6000 m gradient (38 and 29% for experienced men and women runners) (Temesi et al., 2015). The current MVC decreases observed in men are larger than the 25–30% reported after shorter leveled 10 km (Nicol et al., 2003) or marathon runs (Nicol et al., 1991b; Avela et al., 1999). Both men and women MVC decreases are also larger than the 17.9 and 1.8% reported after a 2 h treadmill run (Glace et al., 1998) and the 10% of both men and women after a 20 km running race without gradient (Boccia et al., 2018). At 2H, men’s decrements in MVCs were still large, but close to the ones reported after marathon (Avela et al., 1999) or downhill (Chen et al., 2007) runs. Taken together, these observations emphasize the large acute fatigue induced by the present race profile for recreational men and women runners. Within this framework, our race included a continuous uphill (+382 m) from the 5–10th km that resulted in a 2 km.h^–1^ drop of the mean running velocity for both men and women. This type of effort requires an overall increased activity of the lower limb extensor muscles (Cai et al., 2010) and in turn, leads to increased metabolic fatigue (Giandolini et al., 2016). The subsequent downhill sections (∼6 km with −294 m gradient including a maximum slope of 8.4 and 3.5% on average) may be considered as a moderate running effort (Cai et al., 2010). However, given the lack of specific downhill training for most of our recreational runners, the succession of prolonged uphill and downhill sections may have increased the occurrence of muscle damage due to the metabolic fatigue state of the lower limb muscles while working eccentrically. As suggested by Fridén and Lieber (1992), metabolic fatigue is likely to result in inhomogeneous sarcomere resistance to stretch leading to muscle damage. In this regard, our previous fatigue study (Nicol et al., 2003) revealed higher serum creatine kinase activity and greater functional decreases in the delayed recovery phase in participants with previously high acidosis (reflected by elevated serum lactate concentration) at the time of eccentric muscle actions. In the current study, this agrees with the large acute and 4D delayed recovery of the men runners in MVC and DJ.

Regarding the other tests, the DJ revealed for both men and women at POST current temporal and kinetic changes reflecting fatigue-induced loss of resistance to ground impact and deteriorated stretch-shortening cycle type performance (Nicol and Komi, 2010). In the delayed recovery phase, a deteriorated DJ performance was still reflected by reduced velocity and power during the push-off phase while no change was found in the mean force during the braking and push-off phases. As previously reported, polyarticular dynamic tests such as the DJ have the advantage to allow other muscle groups to compensate for the quadriceps femoris fatigue (Nicol et al., 2006; Nicol and Komi, 2010). In the current DJ test, instruction was given to perform it with a short contact time and thus to avoid touching the floor with the heel. This so-called forefoot technique in DJ is reported to rely on a greater use of the gastrocnemii muscles during the braking phase than in the flat foot technique (Cappa and Behm, 2013). Consequently, the major involvement of the triceps surae in the DJ test seems to have compensated for the contractile failure of the quadriceps muscle group, at least during the braking phase. The influence of intermuscular compensation is further supported by the lack of significant decline in the maximal, but pluri-articular, isometric and concentric performances of the HF-V testing protocol. Thus, confirming our expectations, these overall findings warn about the varied conclusions that can be drawn when using a single testing task. As originally emphasized by Bigland-Ritchie and Woods (1984), fatigue may still exist regardless of whether or not a given force or power output can be sustained. The present combination of maximal tests demonstrates that the MVC and DJ tests seem appropriate to detect delayed functional defects, the latter test being likely to require neural compensatory adjustments (i.e., intermuscular compensation). The HF-V and SJ tests may rather reveal unchanged performances while revealing varied muscle activation and/or synergies.

Sex × time interactions were observed on absolute values in the MVC, DJ, and HF-V tests. In the acute recovery phase for the uni- and bilateral MVC tests as well as for the DJ test, the large effect size observed in men, in contrast to the moderate effect size in women, indicates a lesser acute fatigue in women. At POST, the largest drops in performance for male runners resulted in similar absolute levels of isometric force for men and women in the bilateral MVC test. Thus, the well-known statement that men are stronger than women (clearly observed in all PRE-tests) was not systematically observed at the end of the race. This is interesting in terms of the attenuation of the sex difference reported in the performance of longer running races. In the delayed recovery phase, only men showed functional decreases at 2D and 4D in the bilateral MVC test, while no sex difference was found in the unilateral ones. This discrepancy should be explored further in future studies. Regarding the DJ test, the delayed recovery of men was based solely on the decrease of their P_*max*_ at 4D. As reported by methodological studies (Samozino et al., 2008; Morin et al., 2019), this parameter is nevertheless considered the most accurate measure of lower limb explosivity. Take-off velocity (vto), commonly used to evaluate the jump performance, does not take into account changes in jump technics (such as push-off distance and time) or body mass, which may individually change among sessions. Based on these overall observations, the current recreational female runners can be considered as less affected and recovering earlier than their male counterparts. On the other hand, since no sex × time interaction effect was found on the normalized MVC and DJ values, the lesser fatigue effects observed in women may be partially attributed to their lower initial levels of maximal force and power output. Among the underlying mechanisms, women skeletal muscles are reported to have a greater proportional area of slow twitch muscle fibers, which may contribute to their lower power, higher aerobic endurance and lower sensitivity to muscle damage than men (Fridén et al., 1983; Lundsgaard and Kiens, 2014). The lesser acute fatigability of women has also been attributed to the influence of estrogen on muscle perfusion and metabolism (Hunter, 2014, 2016a, 2016b), as well as its antioxidant and membrane-stabilizing effects (Enns and Tiidus, 2010). In the current study, however, the influence of estrogen is attenuated by the lack of homogeneity in the phase of the menstrual cycle of the runners at the time of the experimental protocol.

### Subjective DOMS Sensation

In agreement with our second hypothesis, DOMS were found as being both sex- and muscle-dependent, women reporting higher muscle soreness than men, but only for the hamstring muscle group at 2D. This variability could explain the sex-independent DOMS sensations reported by Oosthuyse and Bosch (2017) after a downhill running, in which the authors did not investigate separately each muscle soreness. On the other hand, our expectation regarding the DOMS disappearance prior to a complete functional recovery was confirmed for the entire population whose triceps surae and quadriceps DOMS disappeared at 2H and 2D, respectively, whereas MVC and DJ were still decreased up to 4D. Confirming the lack of direct relationship between DOMS and functional decrements (Nicol and Komi, 2010), the present DOMS were smaller than the ones previously reported for the quadriceps muscle group after marathon runs leading to comparable delayed functional decrements (Nicol et al., 2003; Kyröläinen et al., 2005).

The major observation was that, despite similar running time and running strategy along the race, only women reported higher DOMS values in the hamstring muscle group. During the second half of the race that consisted mostly of downhills, larger ground impact peaks are likely to have occurred as most runners presented a similar speed than during the first 5 km (level running part) of the race. At that time, their active resistance to ground impact is likely to have been reduced as indicated by their impaired braking phase in the DJ test at POST. Although information is scarce in the literature regarding the hamstring involvement and fatigue in downhill running, Aldret et al. (2017) reported DOMS and a decreased MVC of hamstrings 12 h after a downhill treadmill run. This study included a control group of runners (equipped with an elastic hamstring assistance device) who presented less indirect markers of hamstring damage. Based on the current DOMS sensations, a greater mechanical stress of the hamstring muscles may have occurred in women during the downhill section. In this regard, women are known to have a wider patellar tendon-tibial shaft at all angles of knee flexion (Nunley et al., 2003), which should lead to an increase in the shearing force applied to the hamstring muscle group. The absence of DOMS reported by the male runners for the hamstrings is partly attributed to their greater peak torque and velocity hamstrings/quadriceps ratio (Hewett et al., 2008; Martín-San Agustín et al., 2019).

### Recovery Pattern Specificities

The present work highlights the importance of the multidimensional analyses to reveal interactions between several quantitative and qualitative variables. First, the MFA analysis revealed for the first dimension (explaining 24.03% of the variance) that the DJ and the MVCs were the most effective tests to evaluate the individuals’ recovery profile, while revealing significantly larger decreases for men than for women, especially at POST and 4D. These results reflected the lower functional decrements in DJ and MVCs at POST for women and the remaining deficits for men in DJ at 4D. Importantly, none of the qualitative variables (e.g., DOMS, effort perception) correlated with the MFA dimensions, supporting the absence of relationship with the functional decrements.

The clustering analysis revealed a greater number of recovery profiles than the sole fast and slow recovery profiles classically reported (Nicol et al., 2003; Paulsen et al., 2012). This revealed a panel of recovery profiles along the 4 days (Figure 6). In particular, cluster 6 was mostly composed of women who showed only acute decrements in DJ, emphasizing their rapid recovery. Cluster 2 included the only runner showing large acute and delayed reductions in the pure concentric and pluri-articular HF-V test. Thus, this runner presented an overall decrease in all tests indicating that this participant deserved more time to recover than the others. Cluster 3 included the runners who presented the largest acute decreases in DJ, limited delayed ones, and even some improvements in SJ at 4D showing that their recovery was almost completed. Considering the large inter-individual variability observed in the functional recovery patterns, this analysis presents the major advantage to consider all tests, variables and their variations in the acute and delayed recovery periods, thus characterizing more specifically the individual profiles. The interest of inter-individual analysis has recently been demonstrated by Welch et al. (2020) for the analysis of injured individuals based on their physical performance analysis. This overall analysis is thus considered of importance to define the optimal time for returning to the running practice depending on the recovery phase and on the tests where the decrements were observed. It is interesting to note that when tested a posteriori, this analysis brings additional insight on the potential influence of descriptive variables such as relative race score, effort perception and muscle soreness on the observed clusters. For example, the cluster 4, which reported low values in effort perception after the race, showed delayed decrements in the HF-V test and DOMS up to 4D. This highlights the potential mismatch between the acute subjective perception of exhaustion and the delayed functional decrements.

Some methodological limitations need to be addressed. First, even if this is a methodological limitation commonly encountered in running race studies, the time delay for measuring the fatigue effects at POST (35 ± 15 min on average) may have led to an underestimation of the actual fatigue effects. Further, the limited number of participants (8 men and 10 women) lowered our statistical power. While it was controlled, the involvement of women in different phases of their menstrual cycle may represent a limitation. It should also be noted that, by choosing an ecological race condition, the maximal metabolic involvement may not have been reached by each of the runners. Nevertheless, differing from the review of Deaner et al. (2015) showing that women may take lesser risks than men in marathon races, we did not find any sex difference neither in pacing strategy nor in effort perception. Moreover, the absence of the usual sex-difference in the racing performance may suggest a relatively higher training level for the women’s group. However, no sex-difference was still found on the relative performances once normalized by the winner’s running time for each sex. Finally, based on the improvements observed in the SJ and HF-V tests at 4D, a longer familiarization phase should be used for unusual tests in endurance runners.

## Conclusion

According to our knowledge, this is the first graded running study using an experimental ecologic setting to describe up to 4 days the functional recovery pattern of women vs. men. Supporting our first hypothesis, the functional recovery pattern was found as testing task and sex dependent. Women showed an earlier functional recovery than men in the knee MVC_*BLT*_ and DJ tests. Supporting our second hypothesis, only women reported DOMS for the hamstring muscle group while, for both men and women, DOMS disappeared prior to the complete functional recovery. As expected, the multidimensional analysis was found as a pertinent way to explore the complexity and sex-dependency of the functional recovery pattern. The use of the clustering analyses may thus lead to the identification of runners who may face greater risks of injury when starting to run again. Finally, the analyses based on the normalized functional values emphasize the major influence of the initially higher values of men compared to women.

## Data Availability Statement

The raw data supporting the conclusions of this article will be made available by the authors, without undue reservation, to any qualified researcher.

## Ethics Statement

The studies involving human participants were reviewed and approved by the CERSTAPS (Comité d’Ethique pour la Recherche en STAPS) - 2019-15-09-35. The patients/participants provided their written informed consent to participate in this study.

## Author Contributions

RM, FV, AH, GA, GE, PC, EG, RS, and CN: study conception and design. RM and CN: analysis of data. RM and FV: statistical analysis. RM, FV, and CN: data interpretation and drafting of manuscript. RM, FV, EG, RS, YK, and CN: critical revision. All authors contributed to the acquisition of the data, contributed to the article, and approved the submitted version.

## Conflict of Interest

The authors declare that the research was conducted in the absence of any commercial or financial relationships that could be construed as a potential conflict of interest.

## Abbreviations

MVC: maximal voluntary contraction
SJ: squat jump
HF-V: horizontal force-velocity
DJ: drop jump
IPF: impact peak force (in N)
hpo: distance of push-off (in m)
GRF: ground reaction force (in N)
tpo: push-off time (in s)
t_*brake*_: braking time duration (in s)
SI: asymmetry index (in %)
$\bar{F}$_*brake*_: mean braking force (in N)
ISOM: isometric
$\bar{F}$_*po*_: mean force (in N)
DOMS: delayed muscle soreness (a.u.)
$\bar{V}$_*po*_: mean velocity (in m/s)
RFD: rate of force development (N/s)
$\bar{P}$_*po*_: mean power (in W)
DL: dominant leg
vto: velocity at take-off (in m/s)
NDL: non-dominant leg
P_*max*_: maximal power output (in W)
BLT: bilateral leg
F_*max*_: peak force (in N)
g: gravity (9.81 m/s^2^).
