# Supplementary material for: Sex Influence on the Functional Recovery Pattern After a Graded Running Race: Original Analysis to Identify the Recovery Profiles
Source: Front Physiol. 2021 Mar 18;12:649396. doi: 10.3389/fphys.2021.649396 (PMC8012843; doi:10.3389/fphys.2021.649396)
Supplement: Supplementary file 1 [file Table_1.docx]

**Table S1.** The 16 best explanatory variables revealed by the multiple factor analysis (MFA) on the first and second dimensions. Cos^2^: representation on each dimension. Impact peak force (IPF); vertical push-off distance in squat jump (Hpo_SJ); maximal power (Pmax); mean force ($\bar{F}$), velocity ($\bar{V}$) and power ($\bar{P}$) of the braking (brake) or push-off (po) phase in drop jump (DJ) or horizontal force-velocity (HF-V) test. 60%, with a resistive load set at 60% bodyweight.

| **Dimension 1 (24.03%)** | | | | **Dimension 2 (16.20%)** | | | |
| --- | --- | --- | --- | --- | --- | --- | --- |
|  | **Correlation** | **P value** | **Cos^2^** |  | **Correlation** | **P value** | **Cos^2^** |
| **POST/PRE** |  | | | **2H/PRE** |  | | |
| $\bar{F}$brake_DJ | 0.83 | <0.001 | 0.69 | Hpo_SJ | 0.70 | <0.01 | 0.49 |
| IPF_DJ | 0.78 | <0.001 | 0.61 | $\bar{F}$po_DJ | -0.59 | <0.05 | 0.35 |
| **2H/PRE** |  | | | **2D/PRE** |  | | |
| Pmax_DJ | 0.82 | <0.001 | 0.68 | Hpo_SJ | 0.78 | <0.001 | 0.61 |
| $\bar{F}$brake_DJ | 0.82 | <0.001 | 0.67 | $\bar{P}$po_HF-V_60% | 0.74 | <0.001 | 0.54 |
| **2D/PRE** |  | | | $\bar{V}$po_HF-V_60% | 0.73 | <0.001 | 0.53 |
| $\bar{F}$brake_DJ | 0.88 | <0.001 | 0.78 | $\bar{V}$po_HF-V_20% | 0.72 | <0.001 | 0.52 |
| Pmax_DJ | 0.84 | <0.001 | 0.70 | $\bar{P}$po_HF-V_20% | 0.69 | <0.01 | 0.48 |
| $\bar{P}$po_DJ | 0.84 | <0.001 | 0.70 | $\bar{F}$po_HF-V_60% | 0.66 | <0.01 | 0.43 |
| $\bar{F}$po_DJ | 0.84 | <0.001 | 0.70 | vto_SJ | 0.67 | <0.01 | 0.45 |
| tbrake_DJ | -0.77 | <0.001 | 0.59 | tpo_HF-V_20% | -0.61 | <0.01 | 0.37 |
| tpo_DJ | -0.65 | <0.01 | 0.42 | **4D/PRE** |  | | |
| **4D/PRE** |  | | | Hpo_SJ | 0.76 | <0.001 | 0.57 |
| $\bar{F}$po_DJ | 0.84 | <0.001 | 0.70 | $\bar{V}$po_HF-V_20% | 0.74 | <0.001 | 0.55 |
| Pmax_DJ | 0.83 | <0.001 | 0.69 | hpo_HF-V_0% | 0.73 | <0.001 | 0.54 |
| $\bar{F}$brake_DJ | 0.82 | <0.001 | 0.66 | hpo_HF-V_20% | 0.70 | <0.01 | 0.49 |
| $\bar{P}$po_DJ | 0.80 | <0.001 | 0.64 | $\bar{P}$po_HF-V_20% | 0.69 | <0.01 | 0.47 |
| IPF_DJ | 0.78 | <0.001 | 0.62 | $\bar{V}$po_HF-V_60% | 0.66 | <0.01 | 0.44 |
| tbrake_DJ | -0.66 | <0.01 | 0.43 |  |  |  |  |
| Taille | -0.61 | <0.01 | 0.38 |  |  |  |  |
| Sex | 0.54 | <0.05 | 0.65 |  |  |  |  |
