# Supplementary material for: Sex Influence on the Functional Recovery Pattern After a Graded Running Race: Original Analysis to Identify the Recovery Profiles
Source: Front Physiol. 2021 Mar 18;12:649396. doi: 10.3389/fphys.2021.649396 (PMC8012843; doi:10.3389/fphys.2021.649396)
Supplement: Supplementary file 2 [file Table_2.docx]

**Table S2.** RV coefficients between the groups revealed by the multiple factor analysis (MFA)

|  | **POST** | **2H** | **2D** | **4D** | **Sex** | **Supplementary group** | **MFA** |
| --- | --- | --- | --- | --- | --- | --- | --- |
| **POST** | 1 |  |  |  |  |  |  |
| **2H** | 0.71 | 1 |  |  |  |  |  |
| **2D** | 0.57 | 0.57 | 1 |  |  |  |  |
| **4D** | 0.48 | 0.54 | 0.72 | 1 |  |  |  |
| **Sex** | 0.41 | 0.15 | 0.20 | 0.31 | 1 |  |  |
| **Supplementary group** | 0.32 | 0.24 | 0.40 | 0.35 | 0.31 | 1 |  |
| **MFA** | 0.80 | 0.82 | 0.87 | 0.84 | 0.32 | 0.39 | 1 |
